# Assessment of the activity of secondary caries lesions with short-wavelength infrared, thermal, and optical coherence tomographic imaging

**DOI:** 10.1117/1.JBO.28.9.094801

**Published:** 2023-01-10

**Authors:** Nai-Yuan N. Chang, Tina Dillas, Yihua Zhu, Daniel Fried

**Affiliations:** University of California, San Francisco, San Francisco, California, United States

**Keywords:** shortwave-infrared imaging, thermal imaging, lesion activity, optical coherence tomography, micro-computed tomography, secondary caries lesions

## Abstract

**Significance**: Leakage in the interfaces between restorative materials and tooth structure allows for fluid and bacterial acid infiltration, causing restoration failure due to secondary caries. Dentists spend more time replacing composite restorations than placing new ones. Previous *in vitro* and *in vivo* studies on enamel and root surfaces using shortwave-infrared (SWIR) and thermal imaging during dehydration with forced air have been promising for assessing lesion activity.

**Aim**: We hypothesized that SWIR reflectance and thermal imaging methods can be used to monitor the activity of secondary caries lesions around composite restorations. The objective of this study was to employ these methods to measure the rate of fluid loss from lesions during dehydration with forced air to assess lesion activity.

**Approach**: Sixty-three extracted human teeth with total of 109 suspected secondary lesions were examined using SWIR and thermal imaging during dehydration. The thickness of the highly mineralized transparent surface layer (TSL) at lesion interfaces indicative of lesion activity was measured by optical coherence tomography (OCT). Micro-computed tomography (MicroCT) was used to further confirm lesion severity and structure. OCT and MicroCT measurements of lesion structure, depth, and severity were correlated with fluid loss rates measured with SWIR reflectance and thermal imaging.

**Results**: TSL thickness measured with OCT correlated with both SWIR reflectance and thermal measurements of rates of fluid loss (p<0.05). Increasing TSL thickness led to decreased permeability of lesions, potentially indicating full lesion arrest at TSL≥70  μm. SWIR performed better than thermal imaging for secondary lesion activity assessment, although both methods performed best on smooth surface lesions.

**Conclusions**: Nondestructive SWIR reflectance and OCT imaging methods are promising for clinically monitoring the activity of secondary caries lesions.

## 1 Introduction

In recent years, the prevalence of using shade-matched and radiopaque dental restorative materials for replacing decayed tooth structure after cavity preparation has led to a marked increase in the formation of secondary caries lesions. Dentists now spend more time replacing failed restorations than placing new ones. Maladaptation of bonding materials to tooth structure permits the microleakage of fluids and bacterial acid, resulting in the demineralization of tooth structure extending beneath the restoration. Clinicians are trained to rely on tactile sensation via the dental explorer and visual inspection to determine whether the decay is active or arrested.^1^ Such methods that rely on texture and color are highly subjective and unreliable.^2^ Often tooth structure is heavily stained, and stain is commonly mistaken for demineralization. A major advantage of shortwave-infrared (SWIR) imaging is that stains do not interfere at wavelengths longer than 1100 nm.^3^^–^^5^ Moreover, tooth-like, radiopaque restorative materials may mask secondary lesions, further complicating their detection and diagnosis with current methods. Accurate assessment of lesion activity, depth, and severity is important for clinicians to decide whether intervention is necessary.

A key indicator that lesions have become arrested due to remineralization is the formation of a highly mineralized transparent surface layer (TSL) on the outer layers of the lesions.^6^^–^^14^ The presence of such a layer greatly inhibits the diffusion of fluids in and out of the lesion. Conversely, active lesions lack such a layer and possess a much higher permeability. Hence, the rate of water diffusion out of the lesion reflects the degree of lesion activity. Effective employment of new optical diagnostic technologies that can exploit changes in the light scattering of sound and carious tooth structure and restorative materials have great potential for diagnosing the present state of secondary lesions.^15^

Previous studies have demonstrated that optical changes due to the loss of water from porous lesions can be used to assess lesion severity and activity with fluorescence, SWIR, and thermal imaging.^16^^–^^22^ We have investigated the use of SWIR reflectance methods to indirectly assess water diffusion rates from lesions because the porosity of the outer layers of active lesions is significantly greater than for arrested lesions.^20^^,^^23^^–^^25^ Sound enamel appears transparent in SWIR wavelengths, whereas early demineralization increases SWIR scattering and reflectance. The incident SWIR light is absorbed by the water in the pores at the lesion surface, particularly at wavelengths such as 1460 and 1950 nm that coincide with water molecular absorption bands, thereby reducing surface scattering and lesion contrast. Water loss from pores in the lesion during lesion dehydration produces a marked increase in reflectivity and lesion contrast. Previous studies have shown great potential for SWIR reflectance imaging at 1950 nm for monitoring carious lesion activity with high sensitivity and efficacy due to the enhanced water absorption.^26^

Temperature changes on tooth surfaces during air drying have been exploited to assess lesion activity via thermal imaging. Previous studies utilizing thermal imaging for assessing the activity of lesions on coronal and root surfaces both *in vivo* and *in vitro* have shown considerable promise.^16^^,^^17^^,^^20^^–^^22^ Measured temperature changes reflect the amount of water that diffuses from pores in the lesion to evaporate from the surface.^20^^,^^27^ In addition, because water tends to pool in gaps and crevices, we postulate that thermal imaging may be able to highlight gaps between restorative materials and tooth structure where there is increased microleakage. Such areas should experience larger temperature drops due to greater water retention in such gaps. In a recent thermal imaging study of root caries lesions, pockets in the gingiva were much cooler than the surrounding tooth structure due to water pooling in such areas.^27^ Therefore, both SWIR and thermal dehydration imaging methods have significant clinical potential for the nondestructive assessment of the activity of secondary caries lesions. Improving caries diagnosis should reduce the unnecessary replacement of composite restorations.

Several studies have demonstrated the utility of optical coherence tomography (OCT) for acquiring high-resolution images of lesion structure and activity *in vivo*.^10^ When used at SWIR wavelengths, OCT is useful in determining whether lesions are active and expanding, partially arrested and undergoing remineralization, or fully arrested and remineralized.^6^^–^^8^^,^^28^^,^^29^ This imaging method resolves the reflectivity of each layer of sound and lesion structures, as well as restorative materials. OCT has also been particularly valuable for the measurement of gaps between restorative materials and tooth structure.^6^^,^^30^ Most importantly, OCT can detect the presence of a transparent zone of high mineral content at the lesion surface formed due to remineralization, which serves as a key indicator that the lesion has become arrested. Such transparent surface zones have been clearly resolved in OCT images of lesions both *in vitro* and *in vivo* on coronal and root surfaces.^9^^,^^10^^,^^27^

The purpose of this study is to develop methods to assess secondary lesion activity using SWIR, thermal imaging, and OCT. We hypothesize that lesion characteristics measured with OCT such as lesion depth (LD), integrated reflectivity (ΔR), and thickness of the formation of the highly mineralized TSL correlate with the lesion permeability (fluid loss rate) and activity measured with SWIR and thermal imaging during dehydration with forced air. In this study, OCT and micro-computed tomography (MicroCT) measurements of lesion structure, depth, and severity were correlated with fluid loss rates measured with SWIR reflectance and thermal imaging.

## 2 Materials and Methods

### 2.1 Sample Preparation

**Fig. 1:**
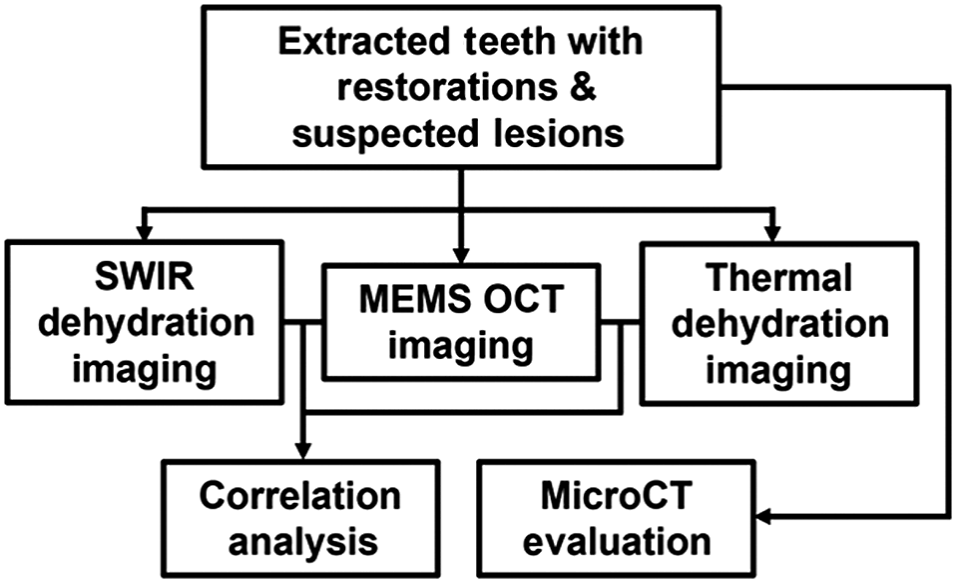
Study workflow schematic.

Sixty-three extracted human teeth collected from oral surgeons were assessed by clinicians for suspected secondary caries lesions. These teeth were sterilized with gamma radiation, mounted into black Delrin blocks using epoxy, and stored in a 0.1% thymol solution. Lesions on each tooth were identified by surfaces per clinical standard, yielding a total sample size of n=109. Figure 1 shows the workflow of the study design.

### 2.2 Visible (Color) Imaging

A USB-powered Model 5MP Edge AM7915MZT digital microscope from AnMO Electronics Corp. (New Taipei City, Taiwan) equipped with eight white LED lights and a visible polarizer to reduce glare was used to acquire 5 mega-pixel (2952×1944) color images of all sample surfaces.

### 2.3 SWIR Reflectance Imaging at 1950 nm

**Fig. 2:**
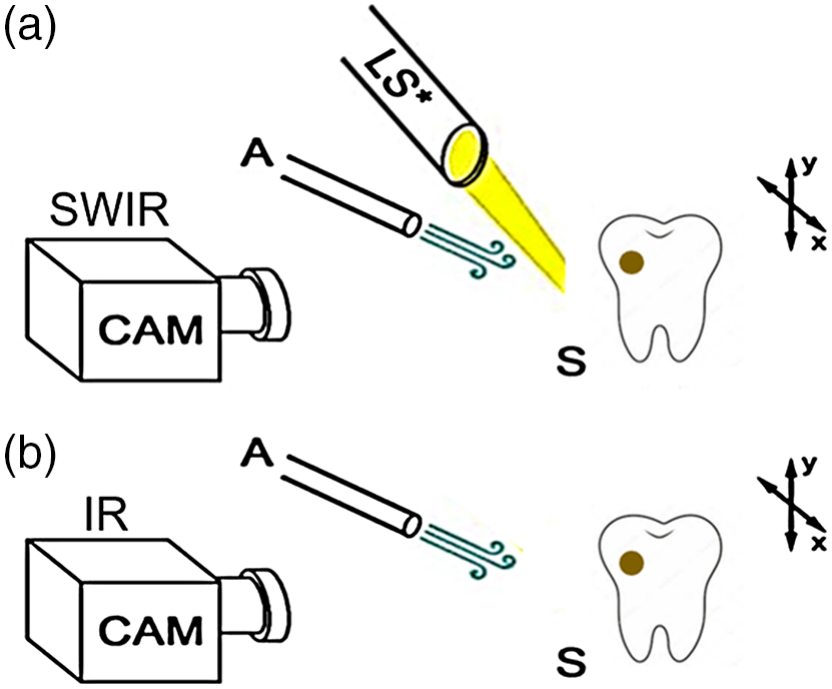
(a) SWIR dehydration setup. (b) Thermal dehydration setup. LS, light source at 1950 nm; A, air nozzle output at 25 psi; F, filter; and S, *ex vivo* sample with suspected secondary lesion.

Samples were stored in a moist environment to preserve internal hydration. Each sample was immersed in a water bath and shaken to enhance water diffusion prior to measurement and then placed into a custom fabricated sample mount. A computer-controlled air nozzle with a 1-mm aperture was positioned 5 cm away from the sample surface at a 20-deg angle. After the sample was removed from the water bath, an image was captured to use as an initial reference image and the air spray was activated. Continuous pressurized air at 25 psi was delivered from the air nozzle to dehydrate the sample as images were captured for 60 s [Fig. 2(a)]. The SWIR reflectance imaging setup was automated using LabVIEW software (National Instruments, Austin, Texas).

A Xeva-2.35-320 extended range InGaAs SWIR T2SL camera (Xenics, Leuven, Belgium) sensitive from 900 to 2350 nm was used to acquire SWIR reflectance images during dehydration. The camera was equipped with two lenses, a Navitar f=35-mm SWIR optimized (f/1.4) lens and a 60-mm achromat lens positioned 40 mm from the 35 mm lens. A high extinction polarizer was used to acquire cross polarization images from 1500 to 2350 nm. The quantum efficiency peaks at 1500 nm near 65%, drops off rapidly to 30% after 1700 nm, and drops off again to below 20% after 2000 nm.

A polarized, broadband amplified spontaneous emission (ASE) light source Model AP-ASE-2000 from AdValue Photonics (Tucson, Arizona) with a center wavelength of 1959 nm, a bandwidth of ∼100  nm (−3  dB), 230 nm (−30  dB), and an output power of 11 mW was used as the 1950 nm light source. The light source was directed at the sample at an incident angle of ∼20  deg to reduce specular reflection, and the source to sample distance was fixed at 5 cm. SWIR reflectance images were processed and automatically analyzed using a dedicated program constructed with LabVIEW and MATLAB (MathWorks, Natick, Massachusetts) software. A region of interest (ROI) encompassing the whole sample was used, and the measurement was recorded for each time point. The mean intensities, ΔIs, were derived from the area under the time–intensity curve

$$\Delta I=\overset{60}{\underset{t=1}{\sum }}(I_{t}-I_{\min})$$

for lesion areas ($\Delta I_{L}$) and control areas ($\Delta I_{C}$). The difference and the ratio between $\Delta I_{L}$ and $\Delta I_{C}$ within the same sample, denoted as $\Delta I_{L-C}$ and $\Delta I_{L/C}$, respectively, were compared to determine which approach is most sensitive to changes in lesion activity.

### 2.4 Thermal Imaging

A similar dehydration setup used for SWIR imaging was used for thermal imaging. A Model A65 infrared (IR) thermography camera from FLIR Systems (Wilsonville, Oregon) sensitive from 7.5 to 13  μm with a resolution of 640×512  pixels, a thermal sensitivity of 50 mK, and a lens with a 13-mm focal length was used to record temperature changes during the dehydration process. The ambient room temperature, flowing air temperature, and water bath temperature were ∼21°C (294.15 K) and were consistent throughout the experiment. The object emissivity was set to 0.92, and the atmospheric temperature was set to 294.15 K.^31^ Although humidity values were not recorded, every sample was measured under the same conditions, with the relative humidity set at a default value of 50%. Previous studies have shown that the area enclosed by the time–temperature curve represents the amount of heat lost, ΔQ, that can be used as a quantitative measurement of the magnitude of evaporative cooling or amount of fluid loss that is indirectly related to the lesion structure and permeability.^16^^,^^17^^,^^20^ Thermal images were processed and analyzed using dedicated programs written in LabVIEW and MATLAB. ΔQ was calculated and recorded as

$$\Delta Q=\overset{60}{\underset{t=1}{\sum }}({\mathrm{Temp}}_{\max}-{\mathrm{Temp}}_{t})$$

for lesion areas ($\Delta Q_{L}$) and control areas $(\Delta Q_{C}$). The difference and the ratio between $\Delta Q_{L}$ and $\Delta Q_{C}$ within the same sample, denoted as $\Delta Q_{L-C}$ and $\Delta Q_{L/C}$, respectively, were compared. Similar considerations as comparing ΔIs warranted us to account for variations in temperature change throughout dehydration in comparing ΔQs.

### 2.5 Optical Coherence Tomography

An IVS-2000-HR-C OCT system from Santec (Komaki, Aichi, Japan) that utilizes a swept laser source and a handpiece with a microelectromechanical scanning mirror and imaging optics was used to acquire complete tomographic images of samples at a volume of 5×5×5  mm. The body of the handpiece is 7×18  cm with an imaging tip that is 4 cm long and 1.5 cm across. Images are captured in ∼3  s at a wavelength of 1312 nm with a bandwidth of 173 nm and a measured resolution in air of 8.8  μm (3 dB). Measured LDs were divided by 1.6, the refractive index of enamel. The lateral resolution is 30  μm ($1/e^{2}$) with a measured imaging depth of 5 mm and depth resolution of 5  μm in air. Image analysis and lesion structural measurements were carried out using Dragonfly from ORS (Montreal, Canada). Using a previously developed processing workflow within the ORS Dragonfly environment, thresholding was performed as image segmentation to estimate the LD, and ΔR was calculated by averaging the intensity values across the ROI. If observed, the thickness of the TSL was also measured.

### 2.6 MicroCT Analysis and Evaluation

A Scanco MicroCT 50 (Wayne, Pennsylvania) located at the UCSF Bone Imaging Core Facility was used to acquire 10  μm resolution images. Image analysis and lesion structural measurements (LD) and surface layer (SL) thickness were carried out also using Dragonfly. A built-in median filter at an appropriate sigma for smoothing and a Sobel-operator for edge detection were used to determine the LD via multiple subsequent measurements using line profiles.

### 2.7 Data and Statistical Analysis

Microsoft Excel (Redmond, Washington) and GraphPad Prism 9 (La Jolla, California) were used for data aggregation, analysis, and graphical presentation. Pearson’s correlation and the student t-test were used for comparing different measurements.

## 3 Results

### 3.1 SWIR Imaging

**Fig. 3:**
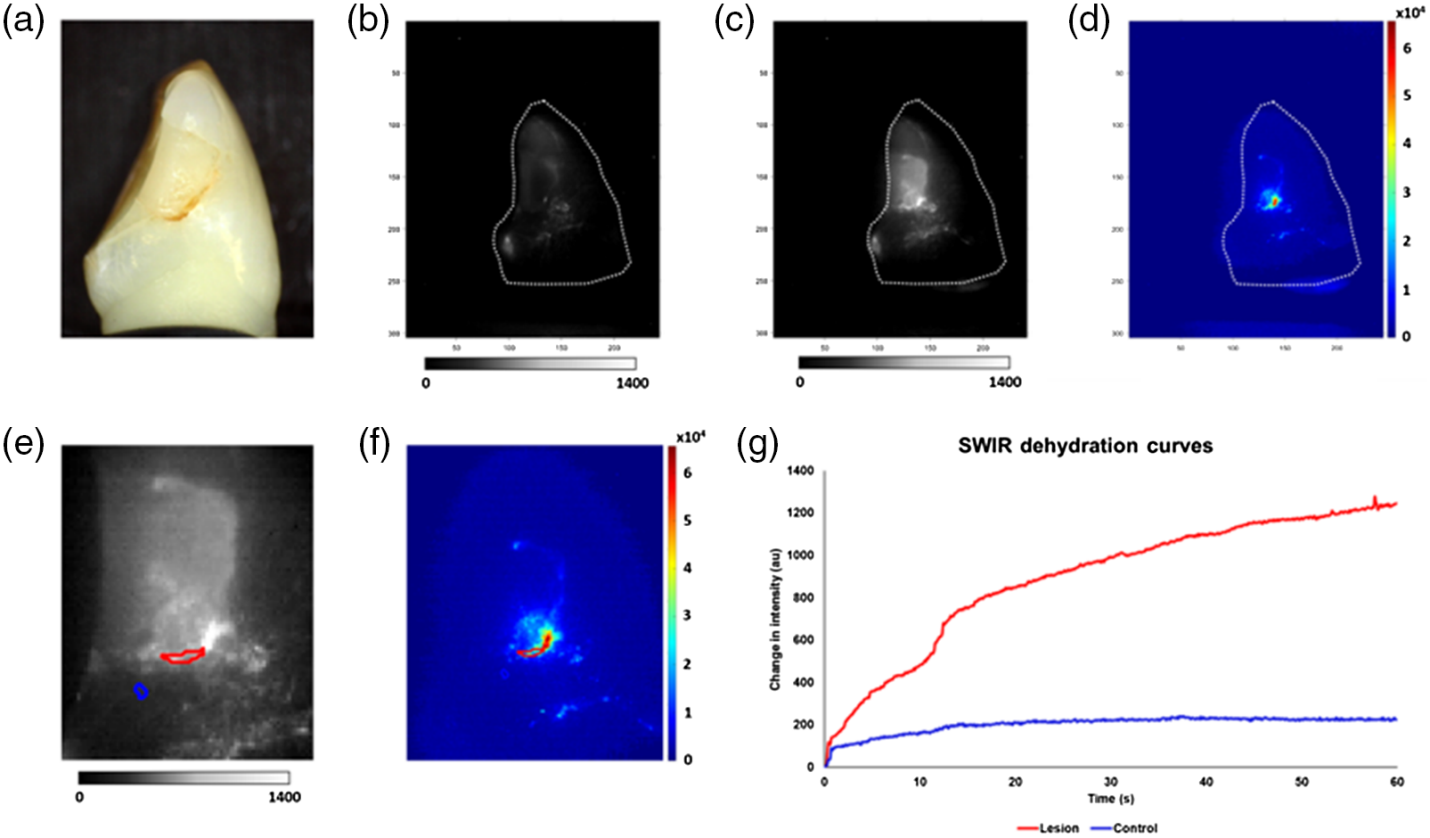
SWIR dehydration analysis results for one of the sample lesions. (a) Visible image. (b) SWIR image at the onset of dehydration. (c) SWIR image at the end of dehydration. (d) Integrated SWIR reflectance intensity change presented as a heat map. (e) SWIR image at the end of dehydration zoomed-in with ROIs (blue = control ROI, red = lesion ROI). (f) Heat map zoomed-in with ROIs. (g) SWIR dehydration curves for control ROI (blue) and lesion ROI (red).

SWIR images acquired during dehydration with forced air of a composite restoration with a suspected lesion are shown in Fig. 3. ΔIs are represented by the areas enclosed by the time–intensity dehydration curves. The integrated heat map allowed the operator to designate the respective control and lesion ROIs. Across all lesion surfaces, the dehydration curves typically exhibited an initial minimum at time zero as when water was present to absorb most of the incident SWIR light. The sigmoidal increase represents both the loss of water absorption and the increase in reflectance of porous areas of demineralization. ΔI values for each lesion were further modified by subtracting the respective control ΔI for each tooth from the lesion ΔI, represented as $\Delta I_{L-C}$, and by dividing the respective lesion ΔI by control ΔI, or $\Delta I_{L/C}$. The $\Delta I_{L-C}$ among the lesions ranged from $1.83\times 10^{4}$ to $3.16\times 10^{6}$ [$6.49\times 10^{5}\pm 6.22\times 10^{4}$ (mean ± SEM)], where $\Delta I_{L/C}$ ranged from 1.10 to 40.3 (5.4±0.6). The SWIR imaging protocol and the collected ΔIs were closely examined to avoid inclusion of any composite restorations, which exhibit high intensities on SWIR reflectance, in the lesion and the control ROIs.

### 3.2 Thermal Imaging

**Fig. 4:**
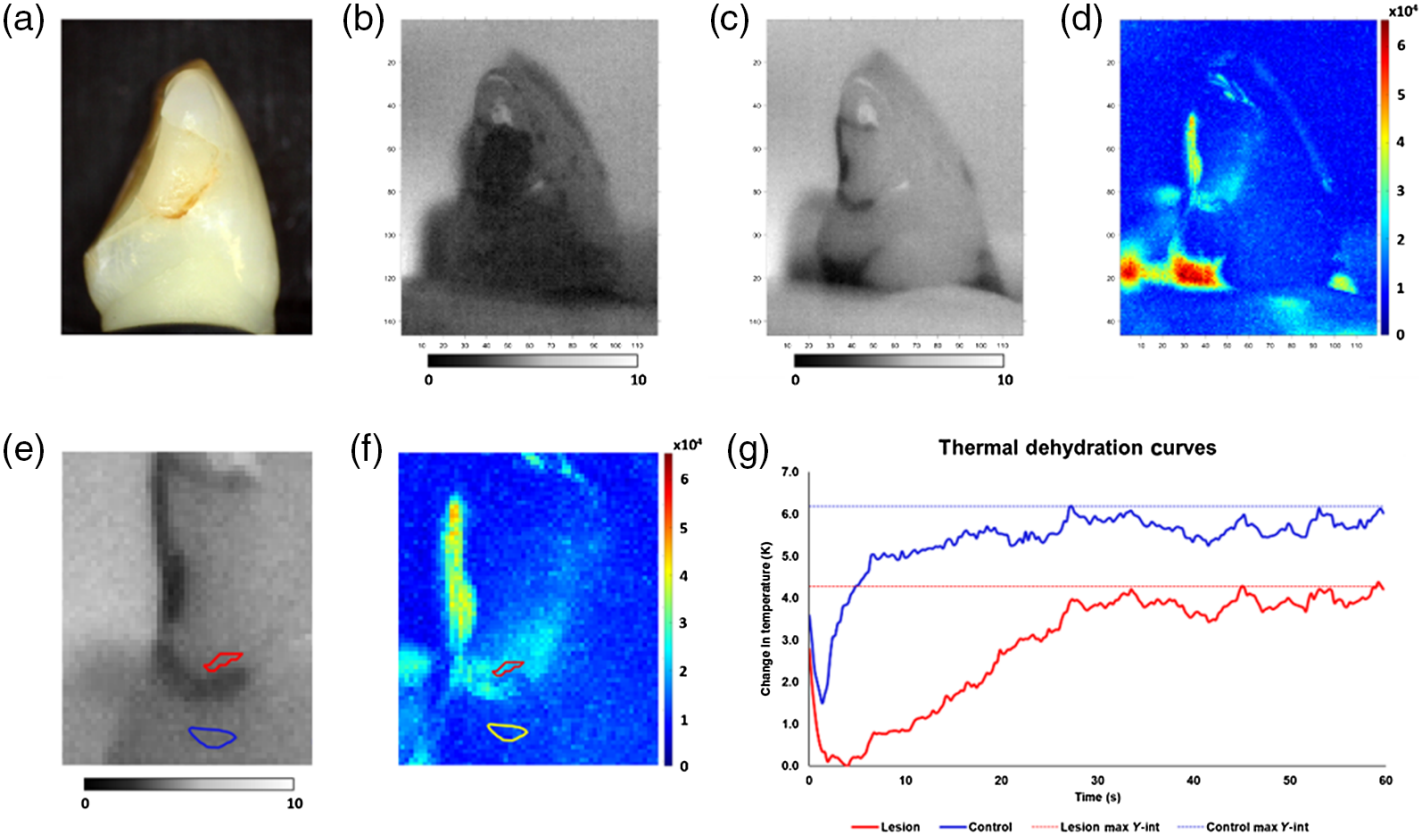
Thermal dehydration analysis results for the same sample lesion shown in Fig. 3. (a) Visible image. (b) Thermal image at onset of dehydration. (c) Thermal image at the end of dehydration. (d) Integrated thermal emissivity change presented as a heat map. (e) Thermal image at the end of dehydration zoomed-in with ROIs (blue = control ROI, red = lesion ROI). (f) Heat map zoomed-in with ROIs (yellow = lesion ROI). (g) Thermal dehydration curves for control ROI (blue) and lesion ROI (red).

Thermal images acquired for the same composite restoration during dehydration are shown in Fig. 4. ΔQ values are represented by the difference between the areas enclosed by the time versus temperature dehydration curves. Across all lesion surfaces, the dehydration curves typically exhibited an initial drop in temperature followed by a slow recovery to the ambient temperature. An overall average dehydration curve was not derived for every lesion as each lesion behaves differently. ΔQ values for each lesion were further modified by subtracting the respective control ΔQ for each tooth from the lesion ΔQ, represented as $\Delta Q_{L-C}$, and by dividing the respective lesion ΔQ by control ΔQ, or $\Delta Q_{L/C}$. The $\Delta Q_{L-C}$ among the lesions ranged from 8 to 617 Ks [193.4±13.4  Ks (mean ± SEM)], where $\Delta Q_{L/C}$ ranged from 1.0 to 6.1 (1.7±0.1). At the onset of dehydration for the sample in Fig. 4, the thermal image showed demarcation between composite material and tooth structure likely because the composite surface was rougher and retained more water after being removed from the water bath. Eventually, the thermal dehydration process was able to overcome the initial water retention and reveal the lesion area, specifically near the interface between the composite material and tooth structure. However, we observed that, for a few samples, the dehydration process was unable to overcome the nonlesion water retention, leading to higher ΔQs.

### 3.3 OCT Imaging

**Fig. 5:**
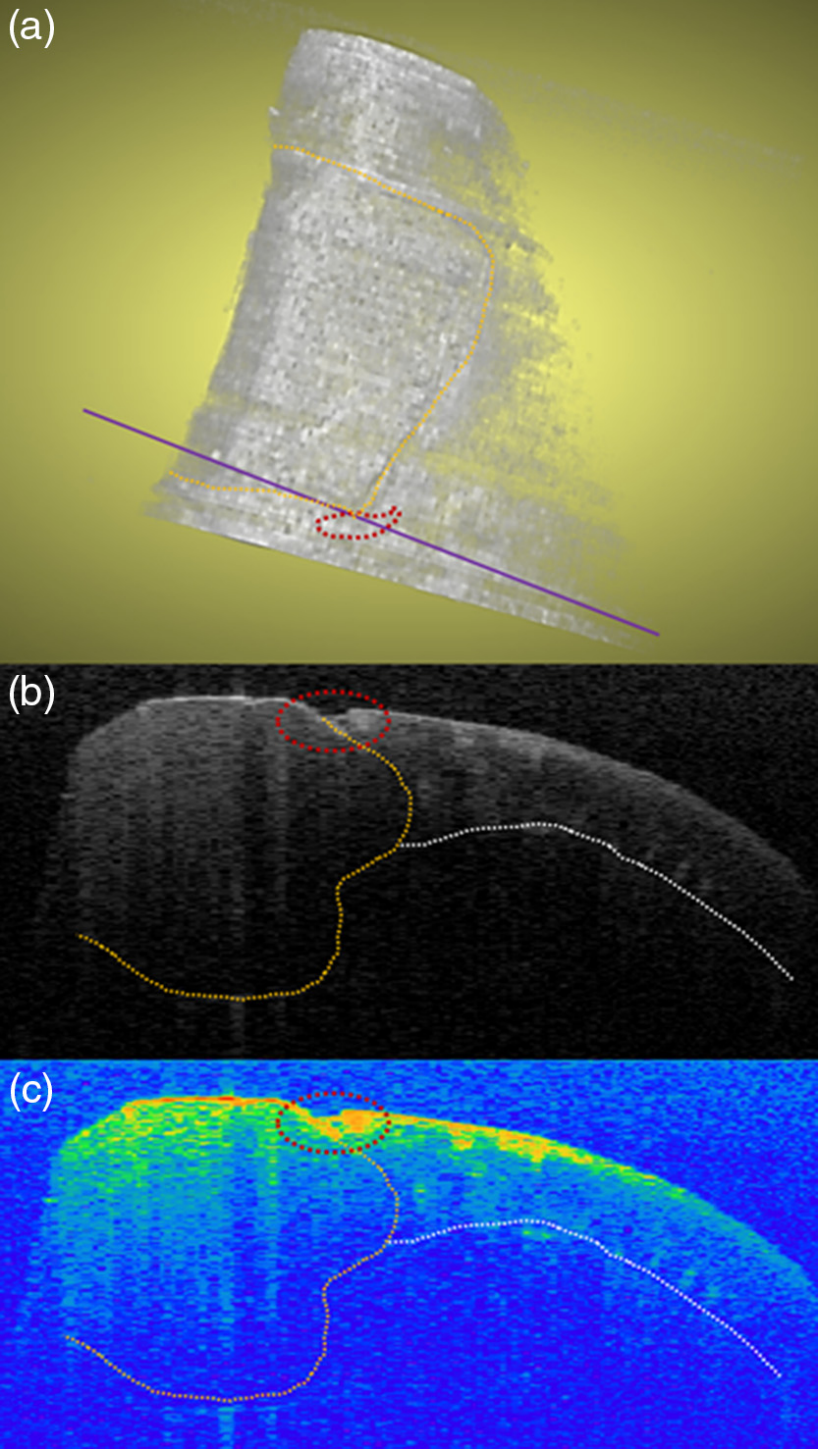
OCT scan of the same sample depicted in Fig. 3. (a) C-scan of the tooth surface with lesion. Orange-dotted area: composite restoration; red-dotted area: suspected secondary lesion area; purple line: location of two-dimensional (2D) B-scan. (b) 2D B-scan of secondary lesion. Orange-dotted line: interface between enamel and dentin; white-dotted line: dentinoenamel junction. (c) 2D B-scan of secondary lesion with threshold segmentation. Note the TSL enclosed by lesion.

Sample OCT C- and B-scans for the same lesion surface are shown in Fig. 5. Estimated LDs ranged from 22 to 340  μm [136±5  μm (mean ± SEM)]. ΔR ranged from 49 to 199 (153±3) for all of the lesions. As expected, ΔR was higher in the lesion area compared with the sound control area. There was a significant positive correlation between LD and ΔR (r=0.24, p<0.05). Of the 109 suspected lesions, 85 lesions had a detectable TSL above or within the lesion, with thicknesses varying from 16 to 100  μm (53±2  μm).

### 3.4 MicroCT Imaging

**Fig. 6:**
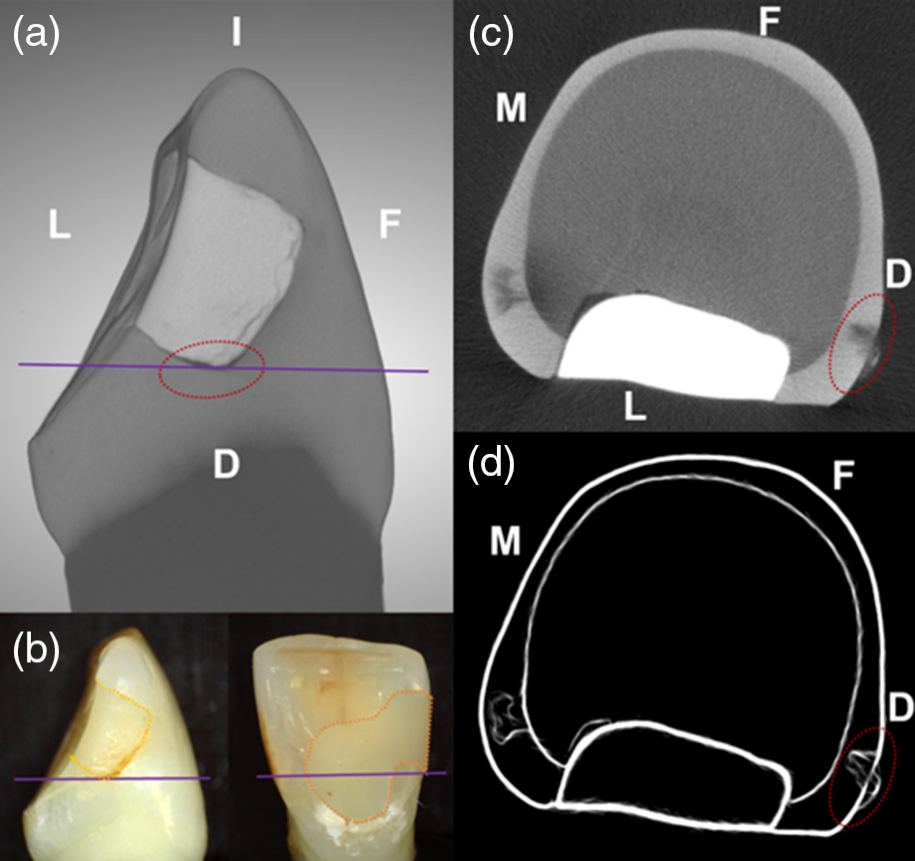
MicroCT scan of the same sample depicted in Fig. 3. (a) 3D scan of the tooth sample oriented to the suspected lesion surface. Purple horizontal line indicates the position of the transverse sectional view. (b) Visible photos of the distal view (inset left) and the lingual view (inset right) of the same sample. Note that the composite restoration (dotted-orange line) extends more apically beyond the level of the transverse section (solid purple line). (c) Transverse slice of the scan at the level of suspected lesion at the purple line position. (d) Transverse slice of the scan at the level of lesion after median smoothing and Sobel-operator edge detection image filtering. Red-dotted oval depicts the suspected lesion area. Surfaces: D, distal; F, facial; I, incisal; L, lingual; and M, mesial.

A MicroCT scan for the same sample shown in Fig. 5 is shown in Fig. 6. Using a previously developed processing workflow within the ORS Dragonfly environment, a combination of a median filter for smoothing and a Sobel-operator for edge detection were used to process the scans to obtain the LD. An SL for each lesion was also recorded. The LD ranged from 57 to 575  μm [235±11  μm (mean ± SEM)]. The SL thickness ranged from 10 to 154  μm (69±2  μm).

### 3.5 Correlation of Thermal and SWIR Imaging with OCT and MicroCT Measurements

**Fig. 7:**
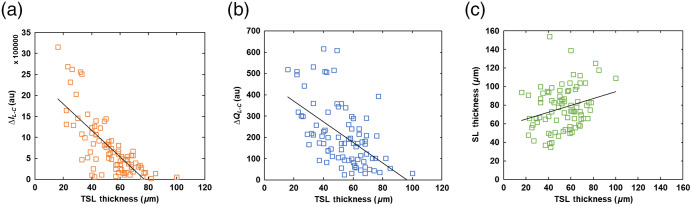
Correlation plots. (a) Between TSL and SWIR dehydration measurement ($\Delta I_{L-C}$) (r=−0.75, p<0.05). (b) Between TSL and $\Delta Q_{L-C}$ (r=−0.54, p<0.05). (c) Between TSL by OCT and SL by MicroCT (r=0.26, p<0.05).

For OCT measurements, there was a significant positive correlation (r=0.24, p<0.05) between the LD and ΔR, i.e., deeper lesions had a higher overall reflectivity. There was no significant correlation between the LD and either thermal (ΔQ) or SWIR (ΔI) measurements. The correlation between ΔR and ΔQ or ΔI appeared to be weakly positive; however, they were not statistically significant. The exception was the correlation between ΔR and $\Delta Q_{L/C}$, in which a significant positive relationship was observed (r=0.23, p<0.05). On the other hand, the TSL thickness was significantly negatively correlated with both dehydration methods; the thicker the TSL was, the lower the dehydration values were (TSL and $\Delta I_{L-C}$: r=−0.75, p<0.05; TSL and $\Delta Q_{L-C}$: r=−0.54, p<0.05; TSL and $\Delta Q_{L/C}$: r=−0.36, p<0.05) [Figs. 7(a) and 7(b)]. The exception was TSL and $\Delta I_{L/C}$, in which the relationship was weakly negative but not statistically significant (r=−0.21, p>0.05). In general, there was no significant correlation between SL as measured by MicroCT and ΔQ or ΔI. However, there was a significant positive correlation between TSL as measured by OCT and SL as measured by MicroCT (r=0.26, p<0.05) [Fig. 7(c)].

**Fig. 8:**
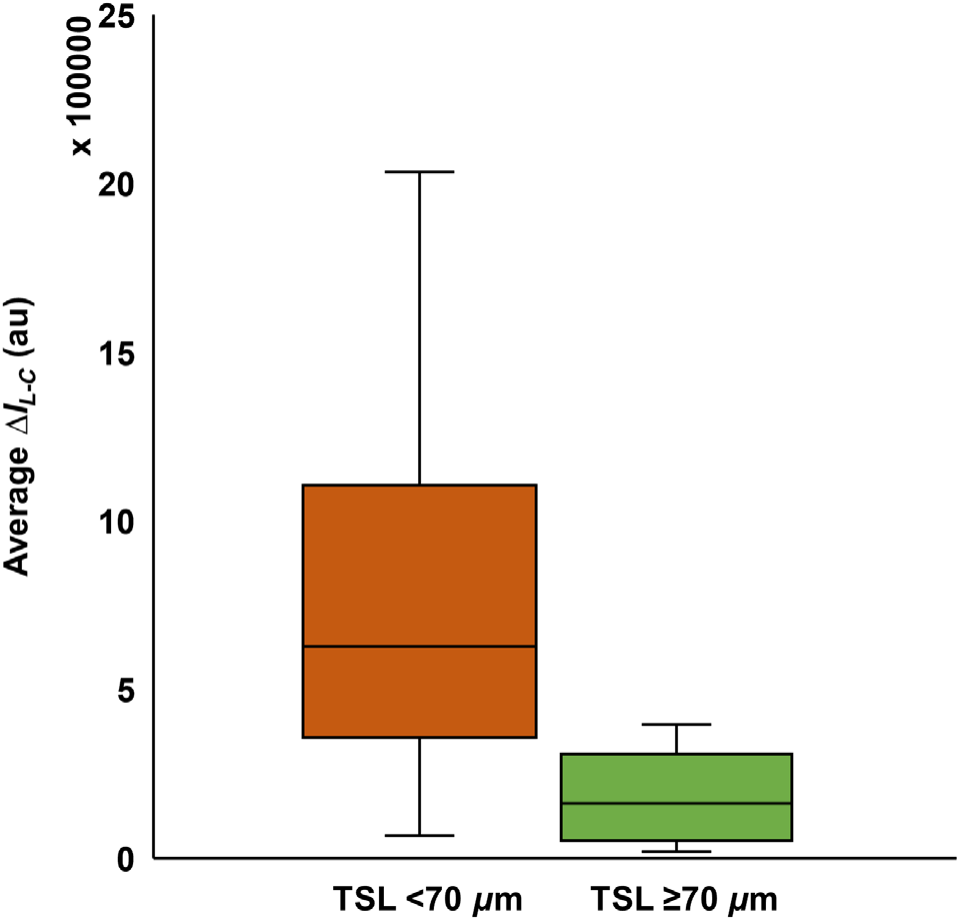
Average SWIR dehydration measurement ($\Delta I_{L-C}$) for lesions with TSL<70  μm and for lesions with TSL≥70  μm (t=2.93, p<0.05).

The influence of the TSL was further examined by investigating the influence of the TSL thickness on ΔQ or ΔI. We found that, for TSL thicknesses <70  μm, there was a significant negative correlation with most dehydration measurements (TSL and $\Delta I_{L-C}$: r=−0.75, p<0.05; TSL and $\Delta Q_{L-C}$: r=−0.52, p<0.05; TSL and $\Delta Q_{L/C}$: r=−0.32, p<0.05) except for TSL and $\Delta I_{L/C}$ (r=−0.145, p>0.05). However, for TSLs of 70  μm or more, there was no further increase in correlation. Unpaired t-tests further substantiated the significant difference between both dehydration values for lesions with TSL of 70  μm or less and those with TSL of >70  μm ($\Delta I_{L-C}$: t=2.93, p<0.05 (Fig. 8); $\Delta I_{L/C}$: t=1.35, p<0.05; $\Delta Q_{L-C}$: t=2.33, p<0.05).

**Fig. 9:**
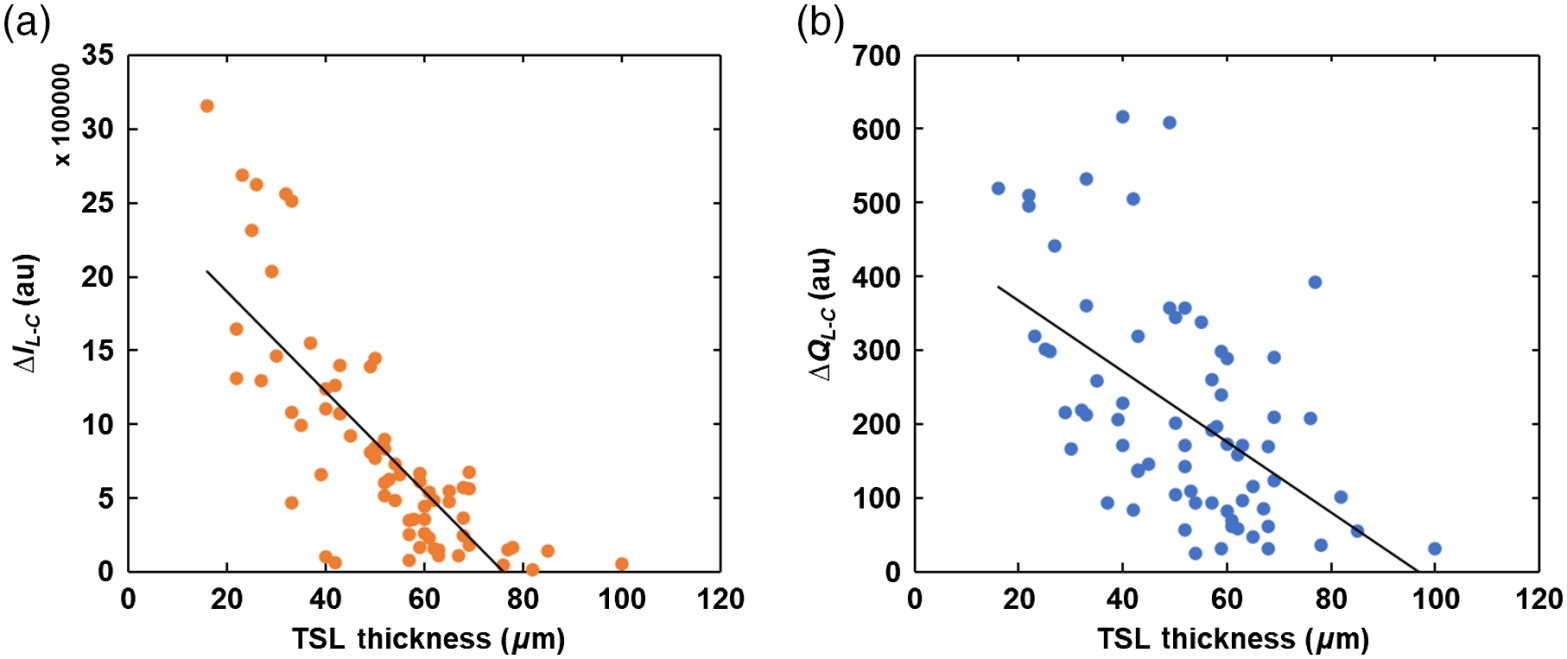
Smooth surface lesions correlation plots between TSL by OCT and dehydration measurements. (a) Between TSL and $\Delta I_{L-C}$ (r=−0.78, p<0.05). (b) Between TSL and $\Delta Q_{L-C}$ (r=−0.54, p<0.05).

We further analyzed the dehydration measurements and the respective OCT measurements based on lesion type: occlusal (n=11), smooth surface (consists of buccal/facial, mesial, distal, lingual; n=69), and incisal (n=5). The correlation analysis showed that there is no significant relationship between the TSL thickness with ΔQ and ΔI for occlusal and incisal lesions. However, there were significant negative correlations of the TSL thickness with ΔQ and ΔI for smooth surface lesions (TSL and $\Delta I_{L-C}$: r=−0.78, p<0.05; TSL and $\Delta Q_{L-C}$: r=−0.54, p<0.05; TSL and $\Delta Q_{L/C}$: r=−0.38, p<0.05) except for TSL and $\Delta I_{L/C}$ (r=−0.22, p>0.05) [Figs. 9(a) and 9(b)].

## 4 Discussion

Several studies have demonstrated the use of thermal and SWIR imaging coupled with dehydration methods to assess the permeability and level of activity of simulated and natural lesions. This study employed similar imaging modalities to assess natural lesions surrounding composite restorations. Previous studies on SWIR reflectance and transillumination imaging of both *in vitro* and *in vivo* secondary caries revealed the capability for SWIR to differentiate between sound tooth structure, lesions, and composite restorations with high contrast.^15^^,^^32^ In this study, SWIR imaging at 1950 nm was advantageous for differentiating composite restorations and lesions from sound tooth structure. Despite the complex geometry and topography in a few lesions, SWIR reflectance imaging during dehydration was able to assess the permeability with relatively close correlation to OCT. However, we did observe, too, that the type of restorative composite material used and the degree of surface roughness have led to a few hyperinflated intensities when no lesions were found in OCT or MicroCT images. Therefore, the accuracy of SWIR dehydration imaging is highly dependent on the user’s ability to carefully designate the ROIs for comparisons between lesion ΔIs and sound ΔIs. Any inclusion of restorative areas in the ROIs may confound the dehydration value calculations and lead to false positives (higher ΔI; determining a lesion as active when it is arrested).

Previous studies have demonstrated the use of thermal imaging for assessing lesion activity *in vitro* and *in vivo*, but this is the first study to investigate its use for imaging secondary caries lesions. An important characteristic of secondary decay is the presence of microleakage at the interfaces between restorative materials and tooth structures. Because areas of increased water pooling and retention yield larger drops in temperature after dehydration, we postulated that thermal imaging is likely to yield information regarding the magnitude of microleakage. Such water pooling has influenced thermal imaging of the pits and fissures of the occlusal surfaces.^17^^,^^21^ In our study, we similarly observed such behavior for occlusal surfaces, around cracks, and over certain restorative materials that appeared to retain more water, resulting in higher ΔQ values when OCT and/or MicroCT images indicated lesion arrest.

Thermal imaging principally detects changes in temperature due to water evaporation, but it inherently does not help differentiate between composite materials and tooth structures due to similar values of thermal emissivity. Differences in the surface texture or composition of some composite materials appear to increase water retention that enables differentiation from tooth structure. We observed that thermal imaging worked well when any pooled water in the lesion area was rapidly removed during dehydration to reveal the lesion area, specifically near the interface between the composite material and tooth structure. In a few samples in which the forced air was not able to rapidly remove any pooled water, false positives (higher ΔQ) were observed. Careful examination of the imaging series was required to ensure the correct designation of lesion and control ROIs to prevent errors in comparisons. Overall, SWIR imaging seemed to outperform thermal imaging in this study. ΔI seemed to be less affected than ΔQ by the complexities of occlusal anatomy and crevices of interfaces that tend to retain water and interfere with thermal dehydration. Thermal dehydration showed greater variability in the measurements compared with SWIR dehydration.

Previous studies have utilized different methods for the analysis of dehydration curves. For SWIR reflectance measurements, we have observed that, although many lesion dehydration curves exhibit sigmoidal growth, several lesions have yielded dehydration curves deviating from this fit. To be more inclusive with our analyses, instead of using the simpler derivation of $\Delta I=\Delta I_{t=0}-\Delta I_{t=60}$,^9^^,^^10^ we chose to integrate the intensity over time. Employing two different ways of comparing dehydration values by calculating differences and ratios between that of the lesion and that of the control allows us to capture more information presented by the complexities of lesion surfaces. Although both differences (L−C) and ratios (L/C) of values between the lesion and the control areas within each sample correlated with TSL thickness, L−C values seemed to correlate more closely than L/C. L/C normalizes and potentially masks portions of the dehydration curve that are attributed to the differences in permeability values between lesion and that of control areas; hence $\Delta I_{L/C}$ for all secondary lesions did not significantly correlate with TSL thickness, but $\Delta I_{L-C}$ did.

LD did not correlate with either type of dehydration measurement, which was not surprising and has been observed previously.^9^^,^^10^^,^^21^ It is likely that the changes in temperature and reflectivity were limited to areas of the lesion that are near the lesion surface and deeper lesion areas have little influence on the dehydration dynamics. In addition, water loss for deeper lesions may require a longer duration for dehydration than was performed here. Alternatively, high subsurface reflectivity due to variable demineralization throughout the whole lesion might have obscured the true LD, contributing to greater variation in the dehydration values for similar LDs.

Based on previous *in vitro* OCT studies using simulated lesions on flat surfaces, we expected ΔR to decrease with increasing TSL thickness due to increasing remineralization.^6^^,^^28^^,^^33^ Here, the relationship was weakly negative but not statistically significant (r=−0.02, p>0.05). It is likely that variation in the topography of the tooth influences remineralization. There is also larger variation of the lesion structure and orientation. Lesions with larger surface area with less demineralization may have similar ΔRs compared with lesions with smaller surface area but greater demineralization. Another reason might be that SWIR light behaves differently with varying composition of the composite restorative materials. Certain composites with opaque shades have titanium dioxide added as an optical opacifier, which blocks the penetration of SWIR light and can interfere with imaging through the composite. Therefore, the composition and surface roughness of some composites might have interfered with accurate measurement of lesions located underneath the composite material. Even so, previous work has demonstrated the ability of different OCT systems to adequately image simulated secondary lesions beneath different composite types *in vitro*.^30^^,^^34^ Therefore, even though surface textures, composite material types, and OCT system types may affect OCT imaging of lesions, there are methods to circumvent such limitations and allow OCT to capture the status of secondary lesions nondestructively for clinical assessment of severity and activity. SWIR and thermal imaging dehydration rates also seemed to correlate more closely with TSL thickness for smooth surface lesions than those restorations located on occlusal and incisal surfaces.

There was significant correlation of the SWIR and thermal imaging rates and the TSL thickness measured using OCT. A previous study using simulated lesions indicated that a TSL thickness of <20  μm led to large permeability changes and that thicknesses of >50  μm might completely arrest *in vitro* lesions.^9^ Another *ex vivo* study also showed that coronal lesions with TSLs thicker than 75  μm led to significantly lower permeability, indicative of lesion arrest.^10^ In this study, we found similar comparisons, in which lesions with TSLs >70  μm were significantly less permeable than lesions with thinner TSLs. Based on these results, we hypothesize that further increases in TSL will have limited influence on the lesion permeability. This provides further confirmation of the utility of SWIR and thermal imaging for monitoring lesion activity.

Although the correlation between permeability measurements and TSL thickness measured using OCT was strong, the correlation between permeability and the SL thickness measured using MicroCT was not significant, even though the correlation between the TSL and SL thickness was significant. It is important to emphasize that, although almost all caries lesions possess some surface zone of increased mineral content, not all surface zones are of sufficiently high mineral density to qualify as “transparent” surface zones that have reduced reflectivity in OCT images compared with the reflectivity of the underlying body of the lesion. Previous measurements of light scattering in demineralized enamel at 1300 nm have shown that light scattering increases markedly with up to 15% mineral loss but increases no further with additional mineral loss.^35^ This is consistent with a network of scattering pores in which further mineral loss only serves to connect the pores without creating more scattering centers to increase reflectivity. In MicroCT, almost all of the lesions had an SL of higher mineral content that was higher than the lesion body. OCT is highly sensitive to slight degrees of mineral loss, and we have previously confirmed that TSLs must contain mineral content near equivalent to sound enamel, making TSL thickness a better indicator of lesion activity compared with SL thickness.^4^^,^^11^^,^^22^^,^^36^^,^^37^ A future study to correlate OCT TSL thickness and surface zone of similar mineral content in MicroCT SL may help further delineate the relationship between OCT and MicroCT for imaging carious lesions.

## 5 Conclusion

In this study, we employed SWIR and thermal imaging methods during dehydration to assess the rate of fluid loss (permeability) that can be used as an indirect measure of the activity of secondary caries lesions. Increasing TSL thickness measured with OCT correlated with decreased permeability of lesions, potentially indicating an arrest in activity at thicknesses exceeding 70  μm. SWIR imaging performed better than thermal imaging for the assessment of secondary caries lesions on tooth coronal surfaces. Thermal imaging performed well in identifying crevices between composite material and tooth structure, but at times can be masked by the complex topography of the occlusal anatomy. SWIR imaging did not appear as susceptible to such interference, owing to the ability to differentiate composite materials, tooth structures, and lesions with high contrast. These results further demonstrate the potential of SWIR reflectivity and OCT imaging methods for the clinical monitoring of the activity of secondary caries lesions.
